# The Pattern of Injuries Among Motorcyclists in Fatal Road Traffic Accidents: An Autopsy-Based Cross-Sectional Study at a Tertiary Care Center in Bihar

**DOI:** 10.7759/cureus.102929

**Published:** 2026-02-03

**Authors:** Toshal D Wankhade, Dinil D Nangelil, Ashok Kumar Rastogi, Amit M Patil, Metta Yaswanth Reddy

**Affiliations:** 1 Forensic Medicine and Toxicology, All India Institute of Medical Sciences, Patna, Patna, IND

**Keywords:** autopsy-based study, injury pattern, motorcycle accident, road safety, road traffic accident

## Abstract

Motorcycle accidents are a major health problem in India as well as globally. Out of the total fatal road traffic accidents, the majority of the cases are due to motorcycle accident injuries. Understanding the specific patterns of injuries sustained by motorcyclists in fatal accidents is crucial for designing effective preventive measures and improving road safety.

The present study aimed to analyse the pattern and distribution of injuries among motorcyclists who succumbed to fatal road traffic accidents, with special reference to demographic characteristics, circumstances of injury, and contributory risk factors. This was a cross-sectional, observational, autopsy-based study conducted prospectively at the mortuary of the Department of Forensic Medicine and Toxicology of a tertiary care centre in Bihar. The study included 58 deceased individuals who died as a result of motorcycle-related road traffic accidents and were subjected to medico-legal autopsy during the study period.

The study findings revealed that the majority of fatalities occurred among adults belonging to the economically productive age group, highlighting the significant socio-economic impact of motorcycle accidents. Male predominance in the study population was noted, reflecting greater exposure and risk-taking behaviour. A considerable proportion of accidents occurred during night hours, suggesting the role of reduced visibility, increased traffic load during evening hours, and fatigue after work. Delay in arrival at a tertiary care facility, gaps in adequate pre-hospital care, emergency response systems, and accessibility of advanced trauma care services were frequently observed.

Non-use of protective helmets emerged as a major contributory factor associated with fatal outcomes. Head injury was identified as the most common and critical injury, often acting as an independent cause of death, either alone or in combination with injuries to other body regions. Autopsy findings demonstrated a high prevalence of cranio-cerebral injuries, including skull fractures and intracranial hemorrhages. Associated injuries to the chest, abdomen, and extremities were also commonly observed, reflecting high-energy impact mechanisms.

This is an autopsy-based study, hence it provides valuable insights into fatal injury patterns and can significantly contribute to evidence-based policymaking and prevention of motorcycle-related deaths.

## Introduction

A road traffic accident is any vehicular accident occurring on the roadway [1]. It is said that road traffic accidents are as old as roads. They must have started taking place since vehicles started running on roads. The first recorded death due to a motor vehicle occurred in the United Kingdom in 1896, followed by the second in the United States in 1899 [2].

Motorcycle accidents are a major public health concern, contributing significantly to the global burden of road traffic injuries and fatalities. Worldwide, motorcyclists account for nearly 23% of all road traffic fatalities [3]. In many countries, motor vehicle accidents rank first among all fatal accidents. Every year almost 1.3 million people die from road accidents in the world [4]. In India, as per the National Crime Records Bureau (NCRB) 2023 report, there were 173,826 road fatalities, out of which 79,533 (45.8%) deaths were due to two-wheelers [5]. Motorcyclists are particularly vulnerable on the road due to their lack of physical protection compared to occupants of other vehicles and the high speeds involved in motorcycle crashes [6-8]. Understanding the specific patterns of injuries sustained by motorcyclists in fatal accidents is crucial for designing effective preventive measures and improving road safety. Studies investigating the pattern of injuries among motorcyclists have primarily relied on data from police reports and hospital records. However, autopsy-based studies provide more detail and accurate information on the nature and extent of injuries sustained by the victims. Autopsy examinations can identify injuries that may have been missed during initial evaluations and provide valuable insights into the specific anatomical regions affected. Tertiary care centers, which receive fatal motorcycle accident cases, serve as important sites for conducting autopsy-based studies.

## Materials and methods

This prospective, autopsy-based, cross-sectional observational study was conducted at the mortuary of the Department of Forensic Medicine of a tertiary care center in Bihar after obtaining approval from the Institutional Ethics Committee (approval AIIMS/Pat/IEC/2023/1126). Inclusion criteria for the study population include all motorcyclists who died due to road traffic accidents and were subjected to medicolegal autopsy during the specified study period. The study was conducted over a period of 18 months. A consecutive sampling method was adopted, and all eligible cases received at the mortuary during the study duration were included. The study was conducted on 58 deceased individuals who met the inclusion criteria with a documented history of road traffic accidents involving motorcycles, as mentioned in police inquest reports or from the history narrated by relatives of the deceased.

Motorcyclists included both drivers and pillion riders. Victims of road traffic accidents involving other modes of transport, such as cars, buses, or pedestrians, were excluded from the study. Written informed consent was obtained from the next of kin of all deceased individuals prior to data collection. Data were collected using a pre-designed proforma through detailed postmortem examination findings, police inquest reports, and interviews with relatives of the deceased. Variables recorded included demographic details such as age, sex, residential status, and occupation; various circumstances of the accident, including the time of the accident, helmet use, and delay in receiving medical care; survival interval; detailed injury patterns involving the head and neck, chest, abdomen, extremities, and other body regions; and the final cause of death. All data were systematically recorded and maintained with strict confidentiality. Descriptive statistical analysis was performed to summarize demographic characteristics and injury patterns. Inferential statistical analysis was carried out to assess the association between helmet use and fatal head injury. Variables were analyzed using the Chi-square test, and a p-value of less than 0.05 was considered statistically significant. Ethical principles of confidentiality, anonymity, and data protection were strictly adhered to throughout the study.

## Results

Demographic profile of victims of fatal motorcycle accidents

A total of 58 victims of road traffic accidents were included in the study. The age of the victims ranged from 16 to 85 years. The mean age was 40.1 ± 16.4 years, with a median age of 36.5 years. The most frequently affected age [mode] was 26 years.

The highest number of cases was observed in the 21-30 years age group (17 cases; 29.3%), followed by the 31-40 years group (10 cases; 17.2%) and the 41-50 years group (nine cases; 15.5%). Very few cases were recorded at the extremes of age, with no victims below 10 years and only one case above 80 years. This indicates that young and middle-aged adults constitute the most vulnerable population for road traffic accidents from the present study (Table 1). 

**Table 1:** Age-wise distribution of deceased motorcyclists (N=58)

Age Group (In years)	Number (n)	Percentage (%)
0–10	0	0
11–20	5	8.6
21–30	17	29.3
31–40	10	17.2
41–50	9	15.5
51–60	8	13.8
61–70	7	12.1
71–80	1	1.7
81–90	1	1.7

**Figure 1:**
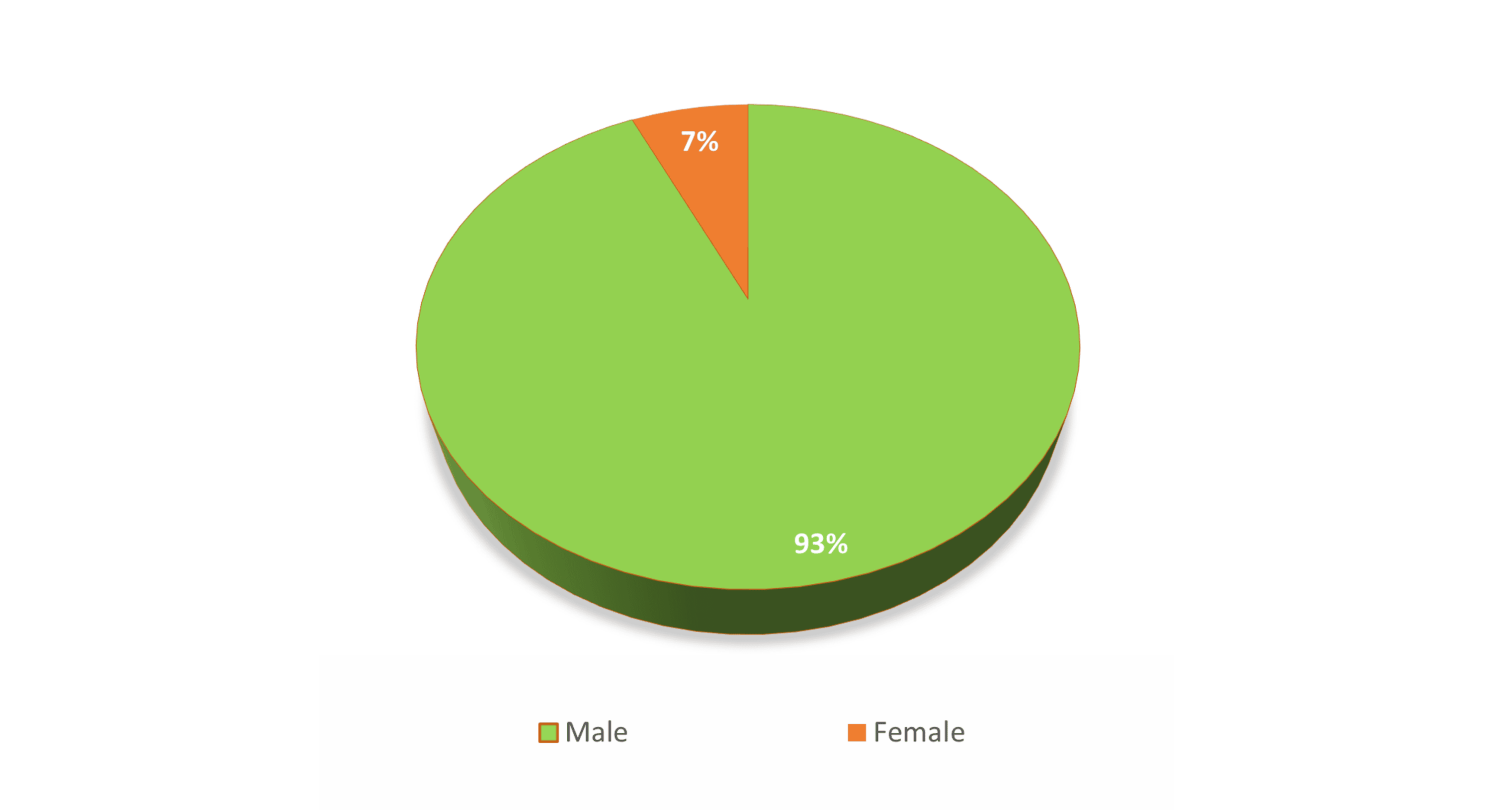
Gender-wise distribution of deceased motorcyclists (N=58)

Considering the gender distribution, out of the 58 victims, 54 (93.1%) were males and four (6.9%) were females, showing a marked male predominance among road traffic accident victims (Figure 1).

Involvement of anatomical location

**Figure 2:**
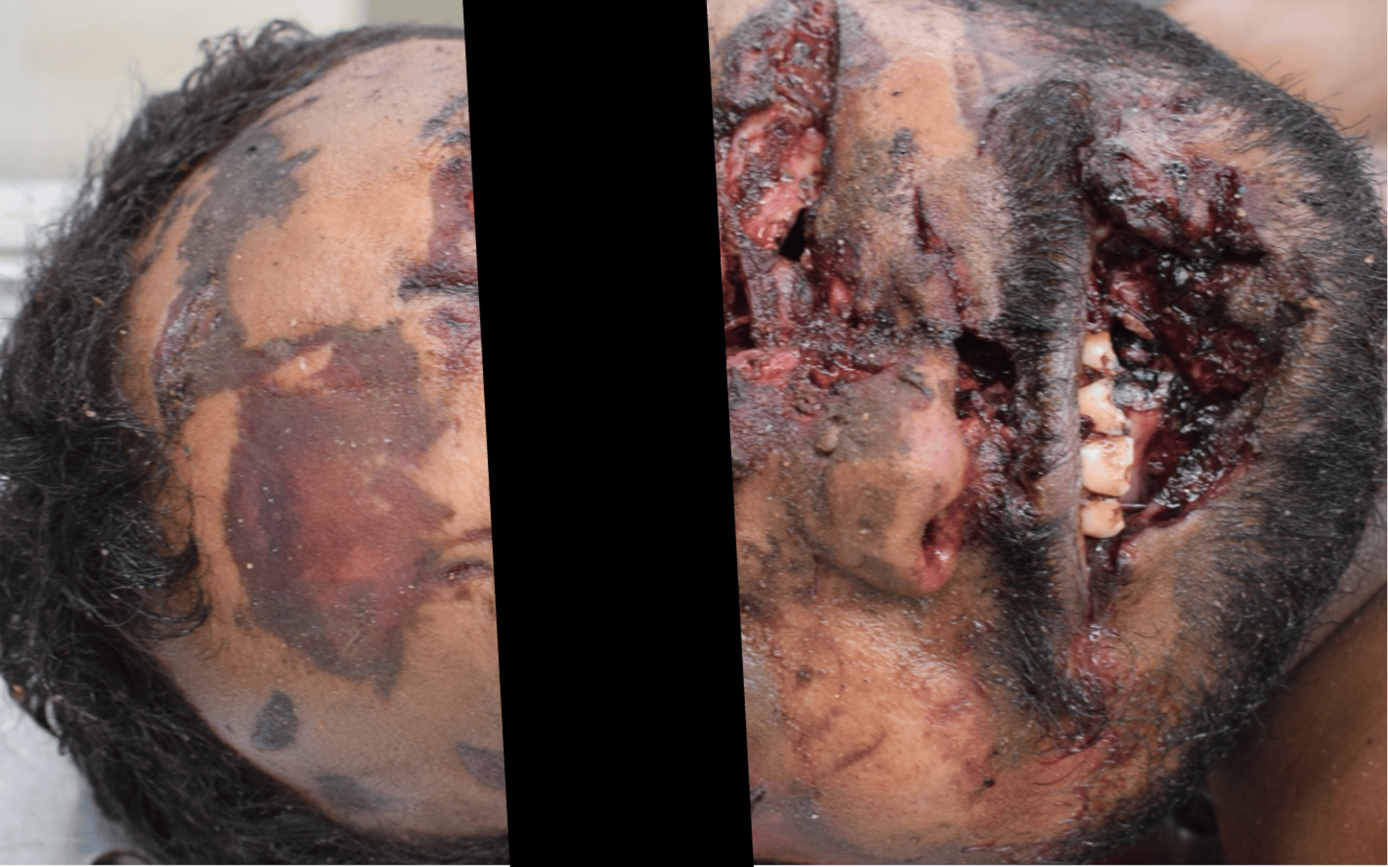
Injury to head and face region

**Figure 3:**
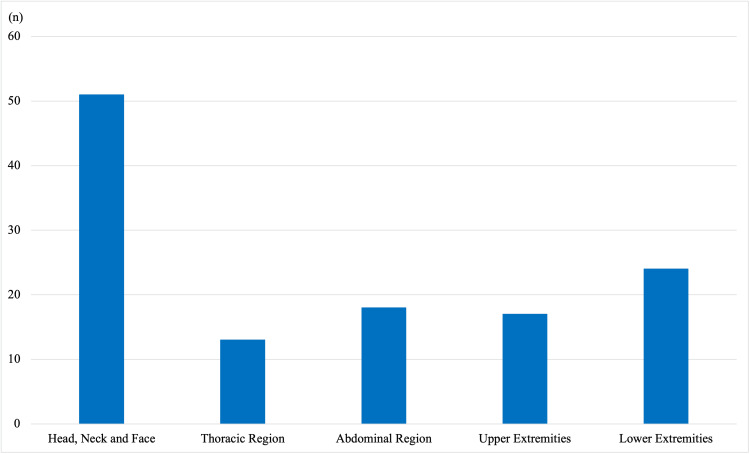
Figure showing distribution of cases as per involvement of anatomical location

Among the 58 victims studied, head, neck, and face injuries were the most commonly involved regions, seen in 51 cases (87.9%) (Figure 2). This was followed by involvement of the lower extremities in 24 cases (41.4%), the abdominal region in 18 cases (31.0%), and the upper extremities in 17 cases (29.3%). Thoracic injuries were observed in 13 cases (22.4%) (Figure 3).

Motorcycle accidents and cause of death

Analysis of the causes of death among the 58 victims of motorcycle accidents showed that head injury was the leading cause, accounting for 49 cases (84.5%). This was followed by abdominal organ injuries in 17 cases (29.3%) and fractures of long bones in 15 cases (25.9%). Chest organ injuries and spinal injuries contributed to death in five (8.6%) and four (6.9%) cases, respectively.

More than one cause of death was identified in 16 cases (27.6%), indicating that a substantial proportion of victims sustained multiple fatal injuries, reflecting the polytrauma nature of road traffic accidents involving motorcycles.

These findings indicate that cranio-cerebral trauma remains the predominant fatal factor in road traffic accidents, while polytrauma and associated musculoskeletal and visceral injuries contribute significantly to mortality (Table 2). 

**Table 2:** Distribution of study samples based on cause of death among deceased motorcyclists (N = 58)

Cause of Death	Number of cases	Percentage (%)
Head injury	49	84.5
Abdominal organ injury	17	29.3
Fracture of long bones	15	25.9
Chest organ injury	5	8.6
Spinal injury	4	6.9
Combination of two or more cause of death	16	27.6

Use of helmets by the victims

Out of the 58 victims studied, only 16 (28%) were using a helmet at the time of the accident, whereas 42 (72%) were not wearing a helmet. This indicates a high prevalence of non-helmet use among road traffic accident victims in the present study. Out of the 58 victims, 49 (84.5%) succumbed due to head injuries.

Among those not wearing a helmet (n = 42), 38 victims (90.5%) had head injuries, whereas among helmet users (n = 16), head injuries were observed in 11 cases (68.8%). This indicates a markedly higher proportion of fatal head injuries among non-helmeted riders. The association between helmet use and fatal head injury was analyzed using the Chi-square test. The Chi-square value (χ²) was 4.18 with 1 degree of freedom, yielding a p-value ≈ 0.04, which is statistically significant (p < 0.05). This demonstrates a significant association between non-use of helmets and fatal head injuries (Table 3).

**Table 3:** Association between helmet use and fatal head injury among deceased motorcyclists (N=58).

Helmet use	Head injury present (n)	Head injury absent (n)	Total	χ² value	df	p-value
Helmet users	11	5	16	4.18	1	0.04
Non-helmet users	38	4	42			
Total	49	9	58			

Pattern of head injuries in victims of motorcycle-related road traffic accidents

Presence of Skull Fractures

**Figure 4:**
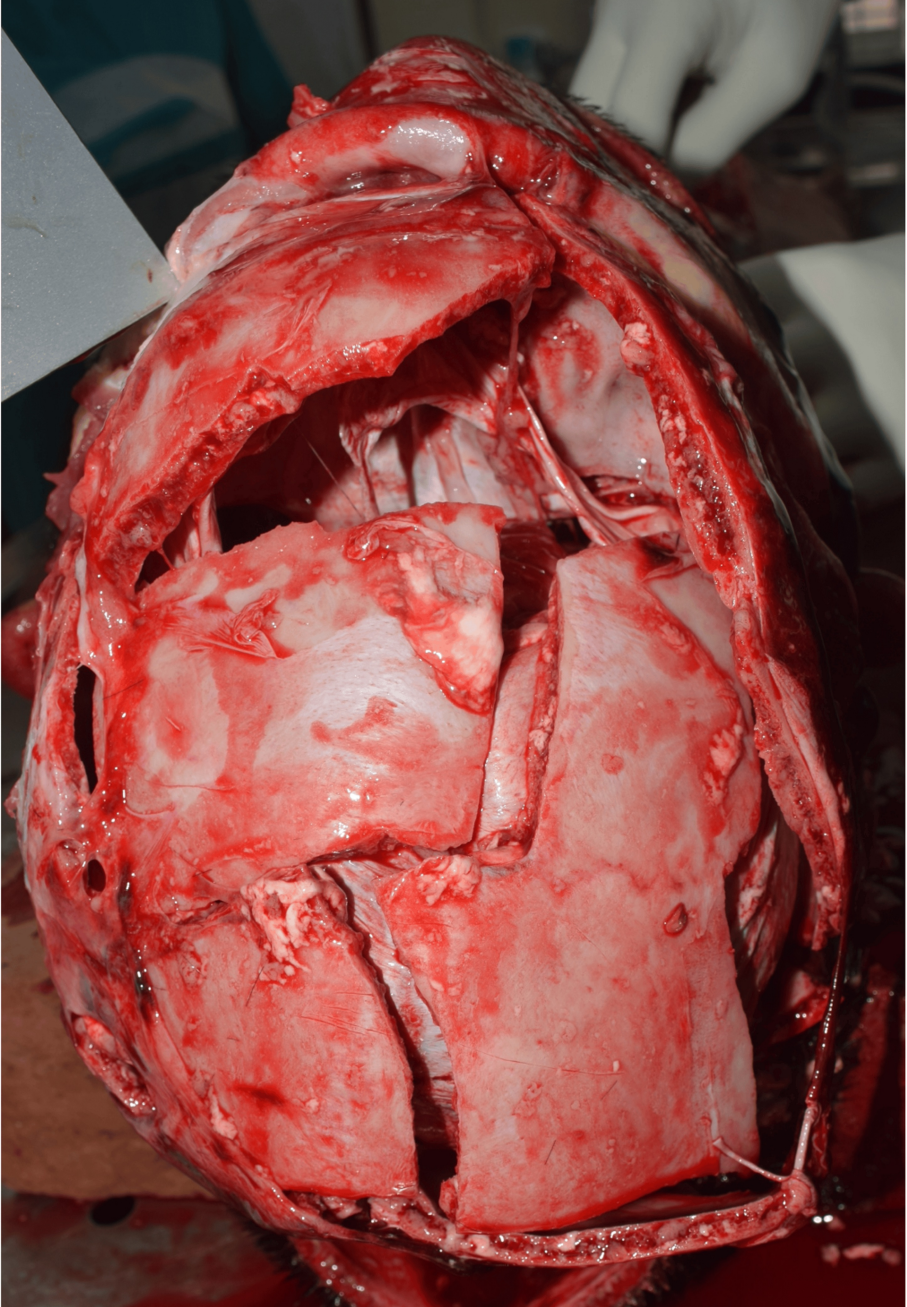
Comminuted fracture of skull

Out of the 58 cases, 36 (62.1%) had presence of skull fractures while 22 (37.9%) showed no skull fracture. Among those with skull fractures, comminuted fractures (Figure 4) were the most frequent (19 cases; 32.8%), followed by linear/fissure fractures (17 cases; 29.3%). Base of skull fractures, comprising anterior, middle, and posterior cranial fossa fractures, were identified in nine cases (15.5%), whereas diastatic fracture was rare, occurring in only one case (1.7%). In several cases (10), more than one type of skull fracture was present, indicating complex injury patterns (Table 4).

**Table 4:** Distribution of cases as per type of skull fracture (N = 58)

Skull Fracture	Number of cases	Percentage
Skull fracture present	36	62.1%
No skull fracture	22	37.9%
Comminuted fracture	19	32.8%
Linear / fissure fracture	17	29.3%
Base of skull fractures	9	15.5%
Diastatic fracture	1	1.7%

Presence of Intracranial Hemorrhages

**Figure 5:**
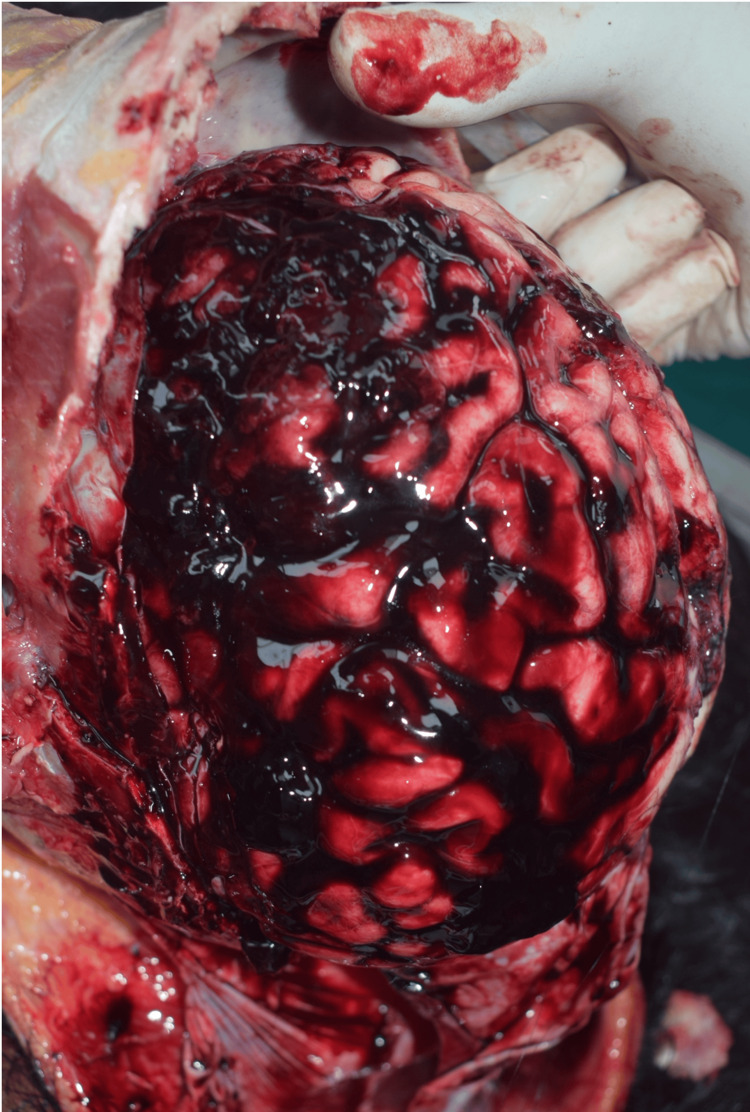
Subdural hemorrhage

**Figure 6:**
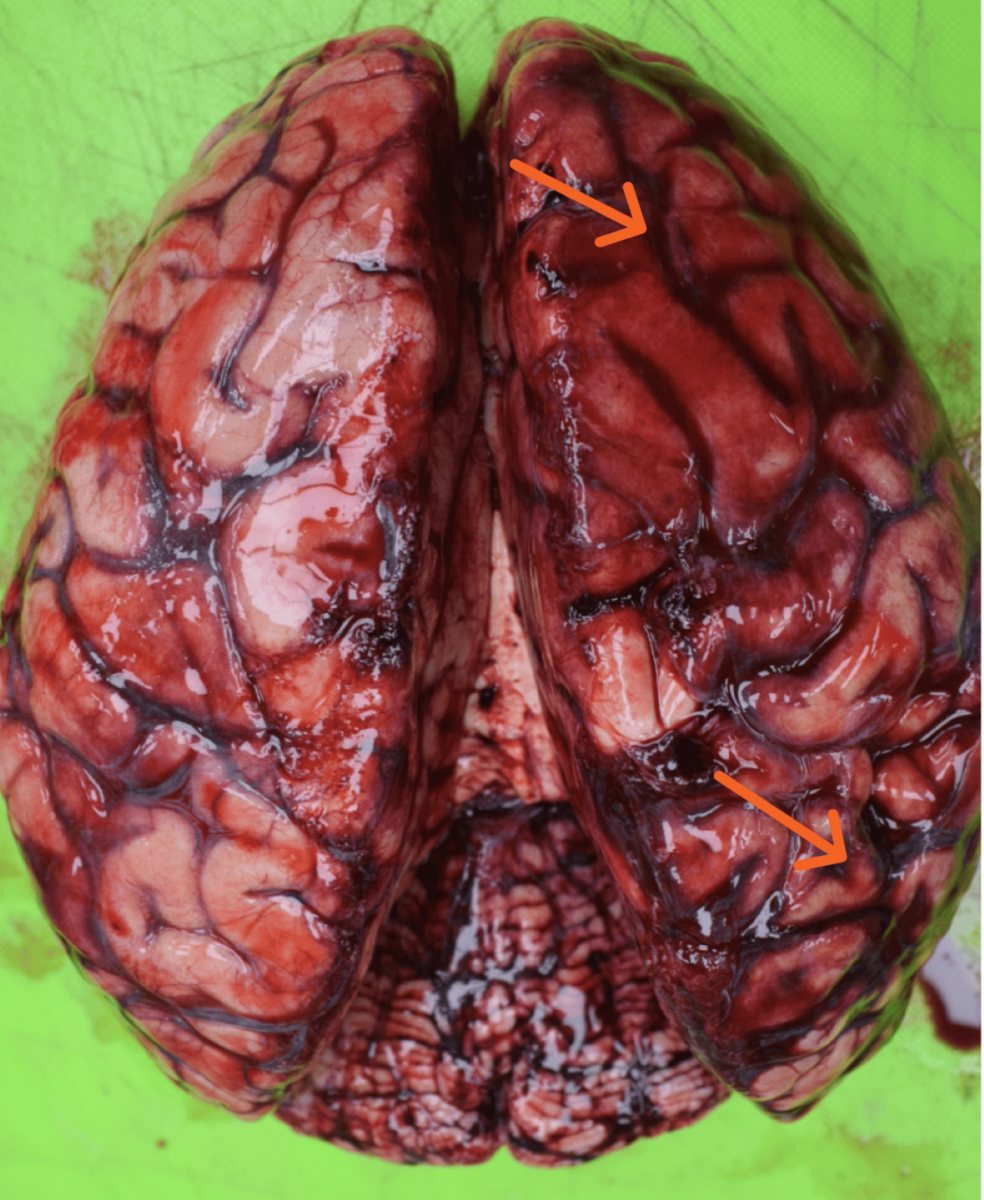
Subarachnoid hemorrhage

Among the 58 fatal motorcycle accident cases, subdural hemorrhage (SDH) (Figure 5) was the most frequently observed intracranial hemorrhage, present in 48 cases (82.8%), followed by subarachnoid hemorrhage (SAH) (Figure 6) in 42 cases (72.4%). Extradural hemorrhage (EDH) was comparatively less common, being noted in only seven cases (12.1%).

A considerable proportion of victims sustained multiple intracranial hemorrhages. The most common combination was SDH with SAH, observed in 35 cases (60.3%), while all three types of hemorrhages (EDH + SDH + SAH) were present in six cases (10.3%).

Nine cases (15.5%) showed no evidence of intracranial hemorrhage, suggesting that death in these victims was likely due to severe injuries to other body parts, further emphasizing the polytrauma nature of fatal motorcycle accidents (Table 5). 

**Table 5:** Distribution of cases as per type of intracranial hemorrhage (N = 58)

Intracranial Haemorrhage	Number of cases (n)	Percentage %
No Intracranial Haemorrhage	9	15.5
Extradural Haemorrhage (EDH)	7	12.1
Subdural Haemorrhage (SDH)	48	82.8
Subarachnoid Haemorrhage (SAH)	42	72.4
SDH + SAH Together	35	60.3
EDH + SDH + SAH Together	6	10.3

Time of occurrence of road traffic accidents involving motorcycles

When the time of occurrence of accidents was analyzed using six-hour time blocks, the majority of incidents occurred during the evening period (06:00 PM-12:00 AM), accounting for 29 cases (50%). This was followed by the afternoon period (12:00 PM-06:00 PM) with 14 cases (24.1%) and the morning period (06:00 AM-12:00 PM) with 11 cases (19%). The night hours (12:00 AM-06:00 AM) recorded the lowest number of accidents, with only four cases (6.9%) (Table 6). 

**Table 6:** Distribution of cases as per time of incident (N = 58)

Time period	Number of cases	Percentage
06:00 AM – 12:00 PM	11	19.0%
12:00 PM – 06:00 PM	14	24.1%
06:00 PM – 12:00 AM	29	50.0%
12:00 AM – 06:00 AM	4	6.9%

Distribution of cases based on time of seeking medical care after the Incident

Out of the 58 victims of motorcycle-related road traffic accidents, eight (13.8%) died at the spot of the accident, and another eight (13.8%) succumbed on the way before reaching the hospital. Among the remaining cases, 29 (50%) victims were brought to the tertiary care hospital within one hour of the incident. Seven cases (12.1%) arrived within two hours, while in six cases (10.3%) the victims were initially taken to a nearby hospital and subsequently referred to a higher center, reaching the tertiary care facility after more than two hours (Table 7).

**Table 7:** Distribution of cases based on time of seeking medical care after the incident (N = 58)

Time to arrival at hospital (Tertiary Care Centre)	Number of cases (n)	Percentage (%)
Died at the spot	8	13.8%
Died on the way before reaching hospital	8	13.8%
Reached tertiary care hospital within 1 hour	29	50.0%
Reached tertiary care hospital within 2 hours	7	12.1%
Initially taken to nearby hospital and reached after >2 hours	6	10.3%

## Discussion

The present study demonstrates a higher involvement of young adults, particularly those in the 21-30 years age group, followed by the 31-40 years age group. This finding highlights that individuals in their most productive years are at the greatest risk of fatal motorcycle accidents. A marked male predominance was also observed among the victims. These observations are consistent with findings reported in several previous studies on road traffic accident fatalities, which have consistently shown higher involvement of young males due to greater exposure to traffic, occupational travel, and risk-taking behaviors [8,9]. Thus, the demographic profile observed in the present study aligns well with the established epidemiological pattern of motorcycle-related fatalities.

In the present study, injuries to the head, neck, and face were the most frequently involved, being observed in 87.9% of cases. This finding is not unexpected, as motorcyclists are highly vulnerable to direct craniofacial impact during collisions due to the absence of structural protection. Similar observations have been reported in several previous autopsy and hospital-based studies, which have consistently identified the head as the most commonly injured and most fatal body region in motorcycle accidents [10,11].

The lower extremities constituted the second most commonly injured region (41.4%), followed by the abdomen (31.0%) and upper extremities (29.3%). Limb injuries in motorcycle crashes are often the result of direct impact with other vehicles, the road surface, or roadside structures, while abdominal injuries commonly occur due to compression against the fuel tank, handlebars, or during ejection from the motorcycle. Thoracic injuries, though less frequent (22.4%), remain clinically significant as they often involve vital organs such as the lungs and heart and may contribute substantially to mortality in high-impact trauma.

These findings underline the importance of enforcing protective measures such as helmet use, protective clothing, and improved road safety strategies aimed at reducing the severity of injuries among motorcyclists.

In the present study, only 28% of the victims were wearing helmets at the time of the accident, indicating a high prevalence of non-compliance with helmet usage. Head injuries were observed in 84% of all victims; however, their occurrence was markedly higher among non-helmet users (90.5%) compared to helmeted riders (68.8%). The Chi-square test demonstrated a statistically significant association between helmet non-use and fatal head injury (χ² = 4.18; p ≈ 0.04). This indicates that helmet use is associated with a reduced occurrence of fatal cranio-cerebral trauma among motorcyclists and supports the protective role of helmets. Similar observations have been reported in multiple epidemiological and autopsy-based studies, which consistently demonstrate a substantial reduction in fatal head injuries among helmet users [10,12,13].

The findings of the present study reinforce the importance of stringent enforcement of helmet legislation and the implementation of targeted road safety education programs to enhance helmet compliance and reduce motorcycle-related fatalities. However, the study also indicates that helmet use does not provide absolute protection, as a significant proportion of fatal head injuries was observed even among helmeted riders. As per Saukko and Knight, although helmets definitely have a protective role, they cannot protect beyond their capacity [14]. Similar findings have been reported in various studies on fatal motorcycle accidents, which show that fatalities occur among both helmeted and non-helmeted riders [8]. However, the incidence of fatal injuries is lower among helmeted users, suggesting the protective value of helmets in motorcycle accidents [10,12]. In addition to severe impact forces exceeding helmet capacity, fatal head injuries among helmeted riders may also be attributed to improper helmet use, such as loose fastening or incorrect positioning, as well as the use of substandard or non-certified helmets.

The present study identified head injury as the leading cause of death among motorcycle accident victims, accounting for 84.5% of cases. This observation is in agreement with previous studies that have consistently reported traumatic brain injury as the most important determinant of mortality in two-wheeler accidents [11-13]. Abdominal injuries, long-bone fractures, chest trauma, and spinal injuries were also common contributors, often occurring in combination and reflecting the polytrauma nature of these accidents. These findings emphasize that although cranio-cerebral trauma is the principal cause of death, associated visceral and musculoskeletal injuries significantly influence fatal outcomes in motorcycle crashes.

The present study demonstrates a high burden of severe cranio-cerebral trauma among fatal motorcycle accident victims. Skull fractures were observed in nearly two-thirds of cases (62.1%), with comminuted fractures being the most common, often occurring in combination with other fracture types, indicating high-energy impact mechanisms. Intracranial hemorrhages were frequently encountered (84.5%), predominantly subdural and subarachnoid hemorrhages, either alone or in combination. The coexistence of multiple intracranial hemorrhages in a substantial proportion of cases further reflects the severity of head trauma sustained in motorcycle accidents. In a smaller subset of cases, the absence of intracranial hemorrhage despite fatal outcomes suggests that death was attributable to severe injuries involving other body parts, highlighting the polytrauma nature of fatal motorcycle accidents. Various literature analyzing injury patterns among motorcyclists have reported findings similar to the present study regarding the distribution of intracranial injuries [14,15]. A study by Slater et al. reported skull fractures in 63.5% of cases, with SDH being the most common intracranial hemorrhage, followed by SAH; however, in their study, fissure fractures were the most commonly observed type of skull fracture [15].

These similarities highlight the consistent pattern of severe cranio-cerebral trauma in fatal motorcycle accidents, as riders commonly fall onto the ground and head injury becomes the most frequent occurrence. 

The majority of accidents in the present study occurred between 6:00 PM and 12:00 AM. Sengupta et al. similarly reported that nearly 70% of fatal road traffic accidents occurred during this time interval [16]. During this period, there is usually heavy traffic on the roads due to office closing hours and people returning home, and in addition, reduced visibility due to diminishing natural light further increases the risk of accidents. Hence the findings emphasize the need for stringent traffic regulations and road safety measures involving the provision of adequate street lighting during these hours.

The present study shows that a substantial proportion of victims died at the spot or during transportation, reflecting the extreme severity of injuries sustained in motorcycle crashes. Although 50% of the victims reached the tertiary care hospital within the first hour (golden hour), survival was still poor, highlighting that early hospital admission alone does not guarantee a favorable outcome when injuries are severe. The study by Gaikwad et al. reported that only 20% of victims reached the hospital within the golden hour and highlighted that injury severity plays a significant role in determining survival than time alone [17]. Similarly, Antony et al. observed that although 55% reached an initial healthcare facility within the golden hour, however the delays in receiving definitive care adversely affected outcomes [18]. Our study shows a significant proportion of early hospital arrival, which reflects improvements in emergency medical services in the study region; however, the persistently high mortality underlines the need not only for rapid transport but also for advanced pre-hospital resuscitation and timely definitive trauma care. The proportion of early arrivals remains lower than that reported from developed countries [19], which indicates existing gaps in trauma management systems and emergency response infrastructure.

In the present study, a subset of victims was initially taken to nearby hospitals and subsequently referred to higher centers because of the severity of their injuries. Such inter-facility transfers certainly lead to delays in definitive management, particularly in cases of severe head and polytrauma. This observation highlights the urgent need for well-equipped and adequately staffed trauma care centers, especially along highways and accident-prone routes, to provide early definitive trauma care and reduce preventable deaths. Strengthening peripheral hospitals with basic trauma life support facilities and establishing an efficient referral and transport network are essential components for improving survival outcomes in fatal motorcycle accidents.

Limitations of the study

This study is limited by its single-center, autopsy-based design which cannot be generalized to entire region-specific data. The present study involved only deceased victims with severe injuries; the protective effect of helmets cannot be fully justified, as the impacts and injuries in these cases were fatal. Information on helmet use and pre-hospital events was derived from secondary sources and may be subject to reporting bias. This study is limited by its single-center, autopsy-based design, and therefore the findings may not be fully generalizable to the wider regional population. The analysis included only deceased victims with severe injuries; hence, the protective effect of helmets cannot be fully quantified, as survivable injury patterns were not represented. Information regarding helmet use and pre-hospital circumstances was obtained from secondary sources, which may be subject to reporting bias. Additionally, detailed data regarding crash dynamics, impact velocity, and helmet quality or fastening status were not consistently available. Indeed, these details can drastically change the potential of the study. 

## Conclusions

Fatal motorcycle accidents predominantly affect young adult males in their most productive years and are characterized by severe cranio-cerebral trauma, frequently associated with non-use of helmets. Head injury remains the leading cause of death, with SDH being the most common intracranial finding followed by SAH. Evening hours constitute the peak period for accidents, and a substantial proportion of victims either die at the spot or during transportation, highlighting the severity of injuries in motorcycle accidents and the importance of rapid trauma care.

The findings emphasize the urgent need for strict enforcement of helmet legislation, promotion of quality and properly fastened helmets, improvement of road lighting and traffic regulation during peak hours, and strengthening of pre-hospital emergency services and trauma care facilities. Encouraging the use of public transport and the development of safer road transport infrastructure by government authorities may further contribute to reducing the burden of motorcycle-related injuries and deaths, as motorcycles represent a highly vulnerable mode of transport. Autopsy-based studies such as the present one provide valuable evidence for formulating effective road safety policies and reducing preventable motorcycle-related fatalities.
